# Rapid Synthesis of Flavor Compound 4-Ethyloctanoic Acid under Microwave Irradiation

**DOI:** 10.3390/ijms11104165

**Published:** 2010-10-25

**Authors:** Yu-Ping Liu, De-Cai Yin, Hai-Tao Chen, Bao-Guo Sun

**Affiliations:** 1 College of Chemistry and Chemical Engineering, Shaanxi University of Science and Technology, Xi’an 710021, China; 2 School of Chemical and Environmental Engineering, Beijing Technology and Business University, Beijing 100048, China; E-Mails: liuyp@th.btbu.edu.cn (Y.-P.L.); ydc8511276@163.com (D.-C.Y.); chenht@th.btbu.edu.cn (H.-T.C.)

**Keywords:** 4-ethyloctanoic acid, microwave irradiation, diethyl malonate, diethyl (2-ethylhexyl)malonate, (2-ethylhexyl)propanedioic acid

## Abstract

Rapid synthesis of 4-ethyloctanoic acid by means of microwave irradiation is described. Diethyl malonate reacted with 2-ethyl-1-bromohexane in the presence of sodium ethoxide to give diethyl (2-ethylhexyl)malonate (**1b**). **1b** was saponified in the solution of ethanol and potassium hydroxide and then acidified to form (2-ethylhexyl)propanedioic acid (**1c**), and **1c** was heated and decarboxylized to give 4-ethyloctanoic acid (**1d**). The influence of reaction temperature and reaction time on the yield of **1b** and the effect of reaction time on the yield of **1c** and **1d** were investigated in order to optimize the synthetic conditions. The relative optimal conditions for the synthesis of **1b** were a mole ratio of sodium to diethyl malonate to 2-ethylhexyl bromide of 0.1:0.11:0.11, a reaction temperature of 80–85 °C, and a reaction time of 2–2.5 h. The yield of **1b** was about 79%. **1b** was saponified for 30 min and then acidified to form **1c**, and the yield of **1c** was 96%. **1c** was heated for 16 min at 180°C to give **1d**, and the yield of **1d** was about 90%. The overall yield of **1d** is 70% under microwave irradiation. The reaction time was reduced greatly. In order to compare the result of microwave irradiation with that of an oil bath, the reactions were also performed in an oil bath. The structures of intermediates, product and by-product were confirmed by HRMS, ^1^H NMR, ^13^C-NMR and IR.

## 1. Introduction

4-Ethyloctanoic acid was first isolated from the root oil of the Costus plant (*Saussurea lappa* Clarke) by de Rijke [1]. In addition to costus root oil, 4-Ethyloctanoic acid also occurs in flue-cured virginia tobacco [2], aged Italian cheese [3], sheep cheese [4], goat cheese [5,6] and stewed beef gravy [7]. 4-Ethyloctanoic acid has waxy, fatty, creamy, moldy, sour sweaty, cheesy odor with animal-like nuances [8] and has been generally recognized as safe (GRAS) flavoring compounds (FEMA No. 3800) and as food additive in USA and China. It can be used for formulating food flavorings and added in meat products, soups, snack foods, milk products, hard candy, and chewing gum to improve their odors [9]. As raw material, 4-ethyloctanoic acid can be used for synthesizing new ester flavor compounds, such as 3-(methylthio)propyl-4-ethyloctanoate, 2-methyl-3-furanthiol-4-ethyloctanoate, 2-furanmethanethiol-4-ethyloctanoate, 4-methyl-5-thiazoleethanol-4-ethyloctanoate [10].

**Scheme 1:**
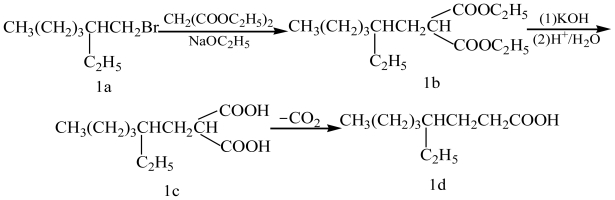
S. G. Powell’s method.

**Scheme 2:**
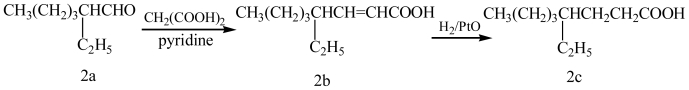
British patent route.

**Scheme 3:**
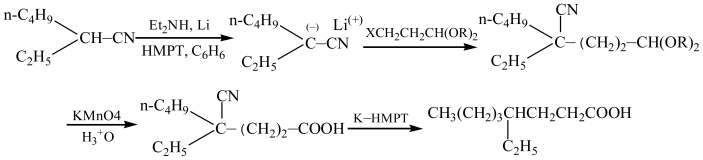
Synthesis by cyano group activation.

**Scheme 4:**
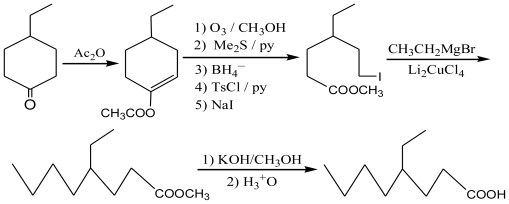
Synthesis by coupling reaction.

4-Ethyloctanoic acid can be synthesized by several methods. One method is that of S. G. Powell’s from 2-ethyl-1-bromohexane and diethyl malonate [11] (Scheme 1). This method offers the advantage of cheap and available materials with mild reaction conditions, but requires long reaction times [12–15]. The British patent route by Knoevenagel reaction [16] (Scheme 2) offers a shorter reaction, but uses more expensive materials. The first step took 20 hours with a 50% yield of **2b**; the second step required platinum oxide to conduct catalytic hydroprocessing under strict operational conditions with an overall yield of 4-ethyloctanoic acid of about 45%. A third method is the preparation of 4-ethyloctanoic acid by using cyano group activation [17] (Scheme 3); these starting materials are not readily available, but an overall yield of 4-ethyloctanoic acid was about 49%, so while this method may be used in small-scale laboratory syntheses and it is not suitable on a larger scale or in a manufacturing plant. The fourth method is the synthesis of 4-ethyloctanoic acid by coupling reaction [18] (Scheme 4). This method includes more steps and complex operational conditions. For example, the coupling reaction requires anhydrous conditions and low temperatures (−70 °C), so this method is only suitable for laboratory syntheses.

Microwave irradiation has attracted scientists’ attention as a powerful tool for rapid, green and efficient synthesis of a variety of compounds [19,20] including synthesis of some FEMA-GRAS approved flavoring agents [21–24]. In view of the advantages and disadvantages of the four synthetic methods, we followed S. G. Powell’s method under microwave irradiation. The aim of the work was to optimize the reaction conditions of each step and supply useful data for a one pot reaction. We also investigated the degree of influence of microwave irradiation on the three chemical reactions. The reaction time was shortened greatly and demonstrating microwave irradiation had the most obvious effect on the alkylation reaction. The reaction conditions of the synthetic steps were optimized in this work.

## 2. Results and Discussion

### 2.1. The Optimization of Synthesis Conditions of Diethyl (2-Ethylhexyl)malonate (**1b**)

We investigated the influences of mole ratio of starting materials, reaction temperature and reaction time on the yield of **1b** to optimize synthesis conditions. Different mole ratios of starting materials were used under the same operational conditions and the results are shown in Table 1. As the ratio of sodium to diethyl malonate increased, the yield of **1b** decreased; as the ratio of 2-ethylhexyl bromide to diethyl malonate increased, the yield of **1b** increased slightly. The reason was that excessive sodium increased the yield of by-product and decreased the yield of **1b**. The by-product was separated by column chromatography and confirmed as diethyl di(2-ethylhexyl)malonate by spectrographic analysis. The by-product was formed by alkylation of **1b**; excessive sodium resulted in the increase of the amount of sodium ethoxide, while sodium ethoxide deprotanated diethyl (2-ethylhexyl)malonate to form a carboanion, which subsequently reacted with 2-ethylhexyl bromide to yield the by-product, decreasing the yield of **1b**. The optimal mole ratio of sodium to diethyl malonate to 2-ethylhexyl bromide was 0.1:0.11:0.11.

The effect of reaction temperature and reaction time on the yield of **1b** under microwave irradiation was investigated. The reaction mixture was sampled and monitored via gas-chromatography at certain intervals. The main results are listed in Table 2. The results show that the higher the reaction temperature, the shorter the reaction time to reach the maximum concentration of **1b**. When the reaction temperature rose from 75 °C to 80 °C, the yield of **1b** increased from 73.3% to 79.1%; however, when the reaction temperature rose from 85 °C to 90 °C, the yield of **1b** decreased from 78.2% to 72.8%. One possible reason is the occurrence of a side reaction. With the rise of reaction temperature, both the rates of the main reaction and the side reaction rate increased, but not to the same extent; this made the yield of **1b** increase first and then decrease. Under microwave irradiation, the optimal reaction temperature was 80–85 °C, and the optimal reaction time was 2–2.5 h. As a comparison, the experiments were performed using an oil bath and the results are shown in Table 3. The results show that when using an oil bath, the reaction takes much more time to reach the higher yield.

The product **1b** was analyzed by ^13^C-NMR. Because **1b** is derived from diethyl malonate, there is a symmetric group in the structure of **1b**, so there should be twelve signals visible in the ^13^C-NMR spectrum; however, there were eleven signals. A chemical shift of C_8_ was the same as that of CH_3_ from OCH_2_CH_3_, and the signal at 14.00 stood for two carbon atoms.

### 2.2. The Optimization of Reaction Time of Synthesis of (2-Ethylhexyl)propanedioic Acid (**1c**)

The reactions were carried out separately in an oil bath and under microwave irradiation in order to compare and optimize the reaction time for synthesis of **1c**, and the results are listed in Table 4. The results indicated that the reaction took a shorter time to reach the higher yield under microwave irritation. The appropriate reaction time for synthesis of **1c** under microwave irradiation was 30 min.

When **1c** was dissolved in absolute ethanol and subjected to gas chromatography to measure the concentration of **1c**, we found that **1c** was decarboxylized to form **1d** at 180 °C. The result suggested that the reaction temperature for preparing **1d** from **1c** would be 180 °C.

### 2.3. The Optimization of Reaction Time of Synthesis of 4-Ethyloctanoic Acid (**1d**)

Because **1c** was decarboxylized to form **1d** at 180 °C, it was not suitable to monitor the reaction via gas-chromatography; however, carbon dioxide is generated in the reaction, so the reaction procedure was monitored by measuring the reducing amount of reaction mixture. The reactions were performed separately using an oil bath and under microwave irradiation and the results are listed in Table 5.

The product obtained under microwave irradiation was slightly more than the product obtained using an oil bath, but there was little difference in reaction time, unlike the above reactions. The reason is that the polarity of **1c** is weak and the reaction is not carried out in polar solvent to simplify the final treatment. The appropriate reaction time for synthesis of **1d** under microwave irradiation was 16 min. After **1c** was decarboxylized to form **1d**, we found the chemical shift of carbon(CH) connected with carboxyl group changed from 49.80 to 38.27 in the ^13^C-NMR spectrums.

The total yields of 4-ethyloctanoic acid on oil bath and under microwave irradiation were calculated; they were 66% (78.9% × 96.1% × 87.0%) and 70% (79.1% × 97.7% × 90.6%). The reactions took more time when using an oil bath; the three steps needed separately 24 h, 150 min and 60 min. However, the three steps needed just 2.5 h, 30 min and 16 min under microwave irradiation.

## 3. Experimental Section

### 3.1. General

Sodium, anhydrous ethanol, diethyl malonate, potassium hydroxide, hydrochloric acid (36.5%), ethyl ether and sodium sulfate (anhydrous) were obtained from Sinopharm Chemical Reagent Beijing Co., Ltd. (Beijing, China). 2-Ethylhexyl bromide was obtained from Jiangsu WDL Chemical Co., Ltd. (Jiangsu, China).

The contents were determined on a Varian CP3800 gas chromatograph. High-resolution mass spectra were obtained at a Bruker Apex IV Fourier-Transform Mass Spectrometry in Peking University. ^1^H-NMR and ^13^C-NMR spectra were recorded on a Bruker DRX-300 nuclear magnetic resonance spectrometer. IR spectra were measured on a Nicolet Avater 370 Fourier transform infrared spectrometer. The reactor was a XH-300A xianghao microwave reactor.

### 3.2. Synthesis of Diethyl (2-Ethylhexyl)malonate **(1b)**

Sodium (2.3 g, 0.1 mol) was dissolved in 40 ml anhydrous ethanol and diethyl malonate (17.62 g, 0.11 mol) was added during agitation. While the resulting mixture was under microwave irradiation, 2-ethylhexyl bromide (21.24 g, 0.11 mol) was added dropwise over the course of 10 min then the reaction mixture was refluxed for 2.5 hours at 80 °C. At the end of reflux, ethanol in the reaction mixture was distilled off and the sample was washed with saturated NaCl aqueous solution. The organic layer was separated, and the aqueous layer was extracted with ethyl ether. The organic layer and the ethyl ether extraction were mixed together, washed with brine, dried with anhydrous NaSO_4_ and filtered to remove solids. After the ethyl ether was evaporated off in *vacuo*, the mixture was weighed, and the content of **1b** in the mixture was determined by gas-chromatography. The product was further purified using distillation under the reduced pressure. B.p. 140 °C/0.8 KPa, HRMS (ESI^+^) m/z calcd. for C_15_H_29_O_4_ [M + H]^+^ 273.20604, found 273.20589, calcd. for C_15_H_28_O_4_Na [M + Na]^+^ 295.18798, found 295.18776. ^1^H NMR (300 MHz, CDCl_3_, *δ*ppm) 4.16–4.22 (m, 4H), 3.41 (t, 1H), 1.82–1.86 (m, 2H), 1.25–1.35 (m,15H), 0.83–0.90 (m, 6H). ^13^C-NMR (300 MHz, CDCl_3_, *δ* ppm) 169.72 (C=O), 61.15 (OCH_2_), 50.04 (CH), 36.71 (CH), 32.48 (CH_2_), 32.35 (CH_2_), 28.50 (CH_2_), 25.45 (CH_2_), 22.91 (CH_2_), 14.00 (2CH_3_), 10.37 (CH_3_). IR (KBr, cm^−1^) 2961, 2931, 2874, 1735, 1464, 1368, 1176, 1150, 1032.

### 3.3. The Isolation of By-Product (Diethyl Di(2-ethylhexyl)malonate) from the Distill Remainder of **1b**

After the **1b** was distilled off, the remaining sample was cooled and subjected to a column chromatographic separation using silica gel and 2% petroleum ether in ethyl acetate. The fractions collected were monitored by thin layer chromatography. The “best” fractions were mixed together, the petroleum ether and ethyl acetate were removed under the reduced pressure, and the purified by-product was obtained as a yellow and transparent liquid. HRMS(ESI^+^) m/z calcd. for C_23_H_44_O_4_Na [M + Na]^+^ 407.31318, found 407.31350. ^1^H-NMR (300 MHz, CDCl_3_, *δ* ppm) 4.09–4.16 (t, 2H), 1.87–1.89 (m, 2H), 1.19–1.26 (m, 12H), 0.77–0.89 (m, 6H); ^13^C-NMR (CDCl_3_, *δ* ppm) 172.79 (C=O), 60.84 (OCH_2_), 56.09 (CH), 36.62 (CH), 34.04 (CH_2_), 33.02 (CH_2_), 28.29 (CH_2_), 25.87 (CH_2_), 23.09 (CH_2_), 14.10 (CH_3_), 13.90 (CH_3_), 10.10 (CH_3_). IR (KBr, cm^−1^) 2958, 2929, 2874, 1731, 1464, 1179, 1380, 860, 727, 558.

### 3.4. Synthesis of (2-Ethylhexyl)propanedioic Acid **(1c)**

Diethyl (2-ethylhexyl)malonate (0.05 mol, 13.6 g) was added to a solution of KOH (11.2 g, 0.2 mol) in ethanol (40 mL 95%). Under microwave irradiation, the reaction mixture was refluxed for 0.5 hour, and then most of ethanol was distilled off and water (100 mL) was added. The resulting mixture was acidified with HCl (conc.) and the organic layer was separated. The water phase was extracted with ethyl ether. The organic phase and extracts were combined, washed with water and brine, dried with anhydrous NaSO_4_ and filtered to remove solids. After evaporation of the ethyl ether, 2-ethylhexylpropanedioic acid was obtained. M.p. 100.5–101.0 °C, HRMS (ESI^+^) m/z calcd. for C_11_H_20_O_4_Na [M + Na]^+^ 239.12538, found 239.12536. ^1^H NMR (300 MHz, CDCl_3_, *δ* ppm) 11.90 (s, 2H), 3.51–3.56 (t, 1H), 1.90 (m, 2H), 1.26–1.35 (m, 9H), 0.84–0.91 (m, 6H). ^13^C-NMR (300 MHz, CDCl_3_, *δ* ppm) 175.86 (COOH), 49.80 (CH), 36.64 (CH), 32.49 (CH_2_), 32.20 (CH_2_), 28.32 (CH_2_), 25.33 (CH_2_), 22.96 (CH_2_), 14.02 (CH_3_), 10.26 (CH_3_). IR (KBr, cm^−1^) 3435, 3006, 2916, 2859, 1604, 1525, 1394, 1374, 1270, 1129, 929.

### 3.5. Synthesis of 4-Ethyloctanoic Acid (**1d**)

Under microwave irradiation, (2-ethylhexyl)propanedioic acid (21.6 g, 0.1 mol) was heated for 16min at 180 °C. The resulting mixture was evaporated in *vacuo* to collect the distillate at 123 °C/0.5 KPa and obtain 4-ethyloctanoic acid. HRMS (ESI^+^) m/z calcd. for C_10_H_19_O_2_ [M – H]^+^ 171.13905, found 171.13894. ^1^H NMR (300 MHz, CDCl_3_, *δ*ppm) 11.67 (s, 1H), 2.30–2.36 (m, 2H), 1.60–1.62 (m, 2H), 1.25–1.31 (m, 9H), 0.87–0.91 (m, 6H). ^13^C-NMR (300 MHz, CDCl_3_, *δ*ppm) 181.05 (COOH), 38.27 (CH), 32.39 (CH_2_), 31.60 (CH_2_), 28.72 (CH_2_), 27.92 (CH_2_), 25.48 (CH_2_), 23.04 (CH_2_), 14.07 (CH_3_), 10.65 (CH_3_). IR (KBr, cm^−1^) 3300-2500, 2960, 2929, 2873, 1711, 1459, 1413, 1380, 1292, 935.

## 4. Conclusions

4-Ethyloctanoic acid was synthesized starting from diethyl malonate through alkylation, saponification, acidification and decarboxylation under microwave irradiation. The reaction of diethyl malonate with 2-ethylhexyl bromide gave diethyl (2-ethylhexyl)malonate in about 79% yield. Diethyl (2-ethylhexyl)malonate was saponified and acidified to form (2-ethylhexyl)propanedioic acid in about 96% yield. (2-Ethylhexyl)propanedioic acid was heated and decarboxylized to give 4-ethyloctanoic acid in about 90% yield. The overall yield of 4-ethyloctanoic acid is 70%. This method provides a simple, rapid and eco-friendly synthesis of 4-ethyloctanoic acid.

## Figures and Tables

**Table 1:** Influence of different mole ratio of materials on the yield.

Run	*n*_(sodium)_	*n*_(diethyl malonate)_	*n*_(2-ethylhexyl bromide)_	Yield of 1b (%)
1	0.105	0.105	0.1	78.1
2	0.110	0.105	0.1	70.6
3	0.110	0.110	0.1	77.3
4	0.115	0.110	0.1	73.4
5	0.1	0.105	0.105	76.1
6	0.1	0.105	0.110	76.6
7	0.1	0.110	0.110	78.7
8	0.1	0.110	0.115	78.8

*Reaction conditions*: reaction temperature, 80 °C; reaction time, 24 h [12]; heating method, oil bath.

**Table 2:** Effect of reaction temperature and reaction time on the yield.

Reaction temperature (°C)	Maximum concentration of 1b in reaction mixture (%)	Reaction time (h)	Yield of 1b (%)
75	70.92	3.0	73.3
80	76.37	2.5	79.1
85	74.22	2.0	78.2
90	70.53	1.5	72.8

*Reaction conditions*: the mole ratio of sodium, diethyl malonate to 2-ethylhexyl bromide, 0.1:0.11:0.11; heating method, microwave irradiation.

**Table 3:** Effect of reaction time on the yield of **1b** in an oil bath.

Reaction time (h)	6	12	18	24
Yield of **1b (%)**	71.0	75.5	77.2	78.9

*Reaction conditions*: the mole ratio of sodium, diethyl malonate to 2-ethylhexyl bromide, 0.1:0.11:0.11; heating method, oil bath; reaction temperature, 80 °C.

**Table 4:** Effect of reaction time on the yield of **1c**.

In oil bath		Under microwave irradiation	

Reaction time (min)	Yield of 1c (%)	Reaction time (min)	Yield of 1c (%)
60	93.0	5	94.3
90	95.5	10	96.1
120	95.9	20	97.4
150	96.1	30	97.7

*Reaction conditions*: the mole ratio of potassium hydroxide to diethyl 2-ethylhexyl-malonate (**1b**), 0.4:0.1; 1b, 0.05 mol; reaction temperature, 85 °C.

**Table 5:** Effect of reaction time on the reaction.

In oil bath		Under microwave irradiation	

Reaction time (min)	The reducing amount of reaction mixture (g)	Reaction time min)	The reducing amount of reaction mixture (g)
5	4.186	4	4.679
15	4.445	8	4.719
30	4.536	12	4.746
60	4.575	16	4.759

Separated yield of **1d(%)**	87.0	Separated yield of **1d(%)**	90.6

*Reaction conditions*: reaction temperature, 180 °C; **1c**, 0.1 mol (21.6 g).
